# *Would* I Trust or *Will* I Trust? The Gap between Entrustment Determinations and Entrustment Decisions for Trainees in Pharmacy and Other Health Professions

**DOI:** 10.3390/pharmacy11030107

**Published:** 2023-06-18

**Authors:** Olle ten Cate, Jennie B. Jarrett

**Affiliations:** 1 Center for Research and Development of Health Professions Education, University Medical Center Utrecht, 3584 CX Utrecht, The Netherlands; 2 Department of Pharmacy Practice, University of Illinois Chicago College of Pharmacy, Chicago, IL 60612, USA; jarrett8@uic.edu

**Keywords:** entrustable professional activity, entrustment decisions making, licensure, autonomy

## Abstract

Entrustable Professional Activities (EPAs) and entrustment decision making are rapidly becoming mainstream in competency-based education in the health professions. EPAs are the units of professional practice to entrust graduates with once they have developed the required competencies. They were conceived to enable a gradual increase in professional autonomy during training, by allowing trainees to practice activities in which they have demonstrated they have mastered well, with decreasing supervision. However, practicing health care unsupervised generally requires licensure. The question for pharmacy education, as well as for undergraduate medical education, is can students be given any autonomy in practice, even when they have fully mastered an EPA yet remain unlicensed? While entrustment decisions for licensed practitioners have autonomy consequences, some educators in undergraduate programs speak of ‘entrustment determinations’, to avoid decisions about students that affect patient care, in other words saying, we would trust you, rather than we will trust you. However, graduating learners without the experience of responsibility and reasonable autonomy creates a gap with full practice responsibilities, which may jeopardize patient safety after training. What can programs do to retain the power of using EPAs while at the same time guarding patient safety?

## 1. Introduction

Entrustable Professional Activities (EPAs) are the novel building blocks increasingly utilized in health professional programs that adhere to competency-based education [1,2,3,4]. More specifically, EPAs are the activities professionals perform that require training, assessment, and permission. Many professionals operate within legally binding rules, regulations, and required licensure to practice. In health professions, protection of patient safety requires such rules, and the quality of patient care benefits from their existence. The question in training is how much autonomy can, and should, learners be given?

Preparing learners for the formal authorization to practice professional activities (whether named EPAs or not) is central to the mission of health professions education, because not only is their goal the awarding of a diploma or degree, it is also to be a gatekeeper of healthcare safety and quality. Most countries have national or state licensing examinations or both, to organize credentialing to practice. The North American Pharmacist Licensure Examination (NAPLEX), for example, is designed to evaluate general practice knowledge in pharmacy and is taken by college of pharmacy graduates in the US once they receive their degree, to attest competence to practice and to provide a standardized test for licensure [5].

## 2. Individualized Development of Learners in the Health Professions

The significance of the use of EPAs in education lies in their potential to operationalize competency-based education (CBE) in the practice of a health profession. With its common use in medicine, CBE is the pursuit of education that qualifies and graduates learners only if they meet the standards of knowledge, attitude, and skill needed for unsupervised practice. Acknowledging that too often a fixed duration in training determines the decision to graduate learners, CBE’s focus is not on time, but on competence. As a license to practice encompasses a broad perspective of professional ability, EPAs break down its breadth into units of practice that can be well overseen, assessed, monitored, documented, and certified [6]. A CBE approach can potentially provide a better indicator that all graduates meet all relevant standards. Yet, learners are not likely to reach the required level of proficiency for each EPA at the same time, and certainly not all at the very end of academic training. Trainees are not identical and their progression towards competence will differ. In addition, much of the training happens in clinical workplaces, and the opportunities for learning across clinical environments (community, hospital, ambulatory care, etc.) vary continuously. For example, the COVID-19 pandemic dramatically converted training opportunities in outpatient practice from in-person to a predominantly telehealth learning experience, providing, among others, a varied mechanism for building communication skills with patients [7]. Yet, many of these learners have graduated with quite different workplace experiences than earlier and later cohorts [8].

## 3. When Are Students Ready to Assume Some Responsibilities and Autonomy?

At the end of a fixed period of time, such as the typical four years of a PharmD program, it is very likely that the readiness for unsupervised practice varies across learners and EPAs. Some may have passed the assessment threshold for unsupervised practice, while others may not yet be ready, speaking to the oddity in the current system that all learners progress to licensing at the same time despite varying levels of proficiency across practice [9]. EPAs serve to create individualized curricula, evaluating learners where they are to make sure that everyone meets competency standards, be it at different moments. Entrustment decisions in EPA based programs can be made informally and ad hoc, by individual preceptors, but eventually a formal qualifying decision for each EPA should be made by an educational team based on an array of information sources that collectively establish the ground to make a summative entrustment decision [10]. In broad health professions, such as pharmacy, this can be challenging, as there are various expertises needed for assessment, such as community and hospital. Pharmacy schools with experiential teams are likely already heading in this direction, when a competency team evaluates learner progress from the range of introductory to advanced pharmacy practice experiences within the curriculum and determining matriculation to more advanced, unsupervised roles. While it may look less like discrete summative decisions of entrustment as in clinical competency committees in medical education, competence is formally evaluated which determines readiness to act unsupervised for each EPA. This is how learners can gradually grow into autonomous practice, step by well-evaluated step, monitored EPA by EPA.

## 4. Scales to Measure Readiness for Entrustment

However, here is where tensions arise. In a discussion of Entrustment-Supervision (ES) scales to be used for EPA-based programs in pharmacy, Jarrett et al. recently noted the “inability to directly observe pharmacy learners complete EPAs that require licensure” as a limitation for full integration of EPAs as an assessment framework [11]. This is more than a practical limitation that can be overcome. It reflects a fundamental issue that plays out differently in different health professions and in different jurisdictions. If pharmacy students cannot be observed in doing what they need to do directly after licensing in practice (not being allowed to do this) then learners, educators, programs, and schools cannot fully attest to their practice readiness. In the past decades, there have been increasing limitations of the autonomy of learners in postgraduate medical [12] and surgical [13] education and training in North America, mostly fueled by concerns over patient safety. Paradoxically, patient safety is jeopardized if graduates do not feel prepared for autonomous practice after graduation and licensure [14,15]. The gradual ‘seniorization’ of tasks in medicine, traditionally done by residents, now by attendings, has alarmed even the Accreditation Council for Graduate Medical Education, as it deprives residents from the experience of autonomous practice while still in a safe training context [16]. These are reasons to consider re-instating more autonomy during training. Interestingly, studies have shown that learner autonomy during training does not necessarily negatively affect patient safety [17].

## 5. Are Entrustment Decisions Always Decisions?

EPAs were originally created for terminal health professional programs, that is, for education leading to the expectation of readiness for unsupervised practice, such as in postgraduate medical specialty training. The adoption of EPAs in undergraduate medical education led to ‘readiness for entering residency training’ [18] and entrustment decisions to focus on working under supervision (be it indirect supervision, i.e., ‘a supervisor quickly available if needed’), rather than unsupervised. Unfortunately, entrustment decisions in many undergraduate medical education programs do not really focus on increased autonomy in the workplace, but just on decisions about progression. Hence, some educators speak of entrustment *determinations* rather than entrustment decisions [19]. Students, being evaluated with Entrustment-Supervision scales, are often rather qualified as ‘we *would* trust this student to work more autonomously’ than ‘we *will* trust (and allow) the student to work more autonomously’. We ‘would trust’ then becomes a powerless qualification, not a decision with impact on responsibility and patient safety, and hard to distinguish from an anchor value on a proficiency scale [20]. It dilutes the power of working with EPAs and feeds confusion about what EPAs are and should be.

Of course, licensure and specialty certification are important considerations that limit the space to allow for autonomy in undergraduate pharmacy education before licensure. Yet, without the experience of autonomy, the potential competency gap between pre- and post-licensure will remain troublesome. Before, as well as after, the legal qualification moment, the context may be adapted to smoothen transitions. Organizing supervision in the first period after licensing and certification is one approach [21,22]. Creating space to explore autonomy before formal licensing or certification by setting the target of meeting the critical objectives of training earlier in the curriculum rather than at the end of the program, while still guarding safety for patients, has also been tried with success [23,24]. 

## 6. Exploring the Space for Autonomy in Undergraduate Programs

Pharmacy is a critical domain of health care and mistakes related to medication use are a dominant cause of error in patient care. It is of the utmost importance to guard quality and safety. However, the solution may not be to provide close supervision and limit autonomy until licensure. One way to counter the risk of graduating and licensing pharmacists who have never experienced autonomy during their training and hence have not been observed with this autonomy, is to set the target of meeting the critical objectives of training, not at the very end of the program, but, say, six months earlier. If framed as a set of EPAs, students should then be monitored to determine whether they can truly be entrusted with these tasks. Decisive moments of entrustment can vary and deviate from the initial plan, but should, after formal entrustment, lead to only marginal checks of student performance and a clear increase in autonomy. Solutions for increased autonomy within the limited regulatory space can sometimes be found creatively, such as ‘watching closely at a distance’ as Babott suggested [25]. For example, a surgical supervisor who has decided not to scrub before entering the OR because she trusts that a trainee is ready for autonomy cannot take over, but is still present and can provide help verbally if needed. Let educators in pharmacy find similar ways to create autonomy for learners when they are ready to bear responsibility after they have demonstrated to be competent, not just because they passed a licensing exam. It is easy to say ‘I would trust’ and not do it, but it is truly relevant to really decide ‘I will trust’, and actually provide the privilege of more autonomy.

## Data Availability

Not applicable.
